# Clinical comparative study of optimized metronidazole loaded lipid nanocarrier vaginal emulgel for management of bacterial vaginosis and its recurrence

**DOI:** 10.1080/10717544.2021.1912211

**Published:** 2021-04-26

**Authors:** Noha M. Badawi, Mona A. Elkafrawy, Rania M. Yehia, Dalia A. Attia

**Affiliations:** a Department of Pharmaceutics and Pharmaceutical Technology, Faculty of Pharmacy, The British University in Egypt (BUE), Cairo, Egypt; b Department of Obstetrics and Gynecology, Faculty of Medicine (Girls), Al-Azhar University, Cairo, Egypt

**Keywords:** Metronidazole, solid lipid nanoparticles, vaginal emulgel, bacterial vaginosis, clinical studies

## Abstract

The main focus of the current work was to design, evaluate and clinically compare the efficiency of novel metronidazole (MTD) loaded solid lipid nanoparticles (SLNs) vaginal emulgel with the marketed vaginal gel (Metron^®^) against Bacterial vaginosis (BV). Eight formulations were fabricated using 2^3^ full factorial design and prepared by stearic acid and tween 80 as solid lipid and surfactant, respectively. Lipid and surfactant concentrations in addition to sonication amplitude were chosen as the independent variables (X_1_–X_3_). Then, the prepared MTD loaded SLNs were evaluated based on the dependent variables which were particle size, polydispersity index, zeta potential, entrapment efficiency, and cumulative % drug release for 24 h (Y_1_–Y_5_). The in vitro release study exhibited a sustained release of MTD from the SLNs up to 24 h. The optimal MTD loaded SLNs showed nanosized particles (256 nm) with EE% (52%), and an acceptable ZP value (−29.5 mV). Also, the optimized MTD-SLNs formulation was incorporated into Carbopol emulgel and investigated clinically for its effect against BV. Clinical studies recorded significant enhancement in therapeutic response of MTD from optimized SLNs vaginal emulgel formulation regarding the clinical treatment (*p* < .05) and low recurrence rate (*p* < .001) against the marketed product. In conclusion, our findings recommend that the fabricated MTD loaded SLNs vaginal emulgel have significant therapeutic effect in terms of BV management over commercially obtainable marketed vaginal gel (Metron^®^).

## Introduction

Bacterial vaginosis (BV) is considered to be a major cause of vaginal discharge in females of childbearing age (Kenyon et al., 2013). BV is a clinical disorder recognized by a change in vaginal flora away from Lactobacillus species; which normally produce the lactic acid and hydrogen peroxide that maintain the acidic pH; toward various bacterial species, including different anaerobes such as *Peptostreptococcus* sp., *Gardnerella vaginalis*, *Mycoplasma hominis*, *Staphylococcus* sp., and the Enterobacteriaceae (Pandey et al., 2020). The altered microbiome causes the production of volatile amines, destroying the protective epithelium layer of both vagina and cervix, leading to a rise in vaginal pH (>4.5) and symptoms that varied from symptomatic to asymptomatic infection (Masoudi et al., 2016). Apart from the discomfort of BV, the case is accompanied with numerous adverse consequences. Furthermore, the existence of other sexually transmitted infections, for example, *Trichomonas vaginalis*, *Chlamydia trachomatis*, *Neisseria gonorrhoeae*, and herpes simplex virus infection seems to be related to an increased occurrence of BV (Esber et al., 2015; Soper, 2020). Moreover, untreated BV is allied with a considerable increase in the risk of HIV attainment (Soper, 2020). It was also stated that women at the age of 40 and above were more vulnerable to BV due to the decrease in estrogen level thus varying the living environment of the *Lactobacillus* sp. (Pandey et al., 2020). Treatment of BV is mostly applied with oral and vaginal formulations of antibiotic drugs such as metronidazole (MTD) or clindamycin that are generally used (Verwijs et al., 2020).

MTD is a drug of choice for the treatment of BV because it has a minimal effect against the normal vaginal flora lactobacilli (Masoudi et al., 2016). MTD is a drug from the nitroimidazole class in which anaerobic bacteria and some parasites; under anaerobic conditions, metabolize it into nitroso radicals, that break microbial DNA’s helical structure, so preventing synthesis of bacterial nucleic acid and finally cause bacterial cell lysis (Verwijs et al., 2020). MTD has been recognized with low levels of antimicrobial resistance due to that that lactobacilli are not sensitive to MTD (Verwijs et al., 2020). Unfortunately, the oral administration of MTD is often accompanied by some drawbacks such as gastrointestinal tract and nausea, while rarely associated with headache, vomiting, anorexia, in addition to the bitter or metallic taste of MTD when taken orally (Brandt et al., 2008). On the other hand, using MTD in the vaginal route is of great importance due to its exclusive, low molecular weight thus providing superior permeation across the vaginal epithelial membrane (Bhowmik et al., 2009). Besides, there are merits in the vaginal route; where the application of drug formulations is done directly in the vaginal cavity for achieving systemic and local action since the vaginal membrane has a dense network of blood vessels for effective drug absorption, large surface area, local effect, self-insertion, high blood supply, and evading the first-pass effect. The vaginal route is mainly used to treat vaginal infections, sexually transmitted diseases, or for contraception and semisolid or solid dosage forms are favored for this route (Kalita et al., 2017).

Nowadays, the level of attention in vaginal drug delivery nanocarriers has remarkably grown as they aid in the drug diffusion through vaginal mucus to attain the underlying tissues, therefore overcome the mucus clearance mechanism (El-Hammadi & Arias, 2015). In addition, nanoparticles, for example, liposomes, solid lipid nanoparticles (SLNs), and nanoemulsions have been established for the delivery of antibacterial drugs and have a significant role in bacterial eradication (Bazzaz et al., 2016). Various studies show that the antibiotics’ encapsulation in lipid-based nanoparticles led to improved antibacterial activity in comparison to the free antibiotics due to concentrating the antibacterial agents at the bacterial biofilm interface and work against colonizing microorganisms (Bazzaz et al., 2016). SLNs are colloidal drug delivery systems that have the ability to deliver lipophilic and hydrophilic drugs over an extended period and can reduce the drug adverse effects by avoiding the environment from direct interaction with the drugs (Mosallaei et al., 2013). SLNs are stable drug carriers upon being used in vivo, in addition, they have noticeable features over conventional drug carriers (Mosallaei et al., 2013).

Improvement of vaginal drug delivery was assisted by mucoadhesive gel formulations. The usage of prolonged-release mucoadhesive vaginal gel was assumed to give several advantages including extended residence time of the formulation at the absorption site because of bioadhesion to the vaginal mucosa, prolonged drug release, enhanced bioavailability, and less adverse effects of drug and eventually enhanced patient compliance (Bhowmik et al., 2009). Carbopol 934 is commonly used as a mucoadhesive polymer in gel formulations due to it forms an adhesive bond between a biological and a synthetic surface (Kalita et al., 2017).

To the best of our knowledge, there is no fabricated formulation of MTD-loaded lipid nanoparticles for the vaginal management of BV. Therefore, our main aim was to formulate and evaluate SLNs loaded with MTD and incorporate in an emulgel dosage form to be applied vaginally for the treatment of BV. Moreover, to clinically investigate the effect of the developed MTD Loaded SLNs vaginal emulgel in comparison with the commonly used market product for the treatment of BV.

## Materials and methods

### Materials

Metronidazole, Carbopol 934 were kindly donated by Egyptian International Pharmaceutical Industries Co., EIPICO, (10th of Ramadan City, Egypt). Stearic acid was purchased from El-Nasr Pharmaceutical Chemicals Co. (Cairo, Egypt). Tween 80 was purchased from Fisher Scientific, U.K. Dialysis tubing cellulose membrane (molecular weight cut off 12,000 Dalton) was purchased from Sigma-Aldrich (St. Louis, MO, USA). All other materials were of analytical grade.

### Design of experiments

The 2^3^ full factorial experimental design was constructed in the current study by the use of Deign-Expert^®^ version 12 (Stat-Ease, Inc., Minneapolis, MN, USA) statistical tool. It was implemented to statistically optimize the formulation variables with the purpose of preparing MTD Loaded SLNs, in order to achieve optimal particle size (PS), polydispersity index (PDI), zeta potential (ZP), high entrapment efficiency percent (EE%), and prolonged cumulative % drug release. Eight different trials were designed; three different independent variables were chosen at two levels according to Table 1: lipid (Stearic acid) concentration (X_1_), surfactant (Tween 80) concentration (X_2_), and sonication amplitude (X_3_). On the other hand, the PS (Y_1_), PDI (Y_2_), ZP (Y_3_), EE% (Y_4_), and cumulative % drug release (Y_5_) were selected as dependent variables. Desirability was used for the optimized formula selection which was subjected for further examinations.

**Table 1.** Independent and dependent variables and their levels for 2^3^ full factorial experimental design.

	Levels	
	−1	+1
Independent variables		
Lipid concentration (X_1_, %)	3	5
Surfactant concentration (X_2_, %)	3	5
Sonication amplitude (X_3_, %)	50	100
	Constraints	
Dependent variables		
Particle size (Y_1_)	Minimize	
Polydispersity index (Y_2_)	Minimize	
Zeta potential (Y_3_)	Maximize	
Entrapment efficiency (Y_4_)	Maximize	
Cumulative % drug release (Y_5_)	Maximize	

### Preparation of MTD loaded SLNs

SLNs loaded with MTD were prepared by hot homogenization followed by the ultrasonication method as described before by Badawi et al. (2018). In addition, blank SLNs (void without adding drug) were prepared using the same conditions for the reason of comparison. The developed dispersions were collected in glass screw-capped tubes and kept in the refrigerator for further studies.

### Characterization of MTD loaded SLNs

#### Particle size, PDI, and zeta potential determination

The average PS, PDI, and ZP of the prepared MTD-SLNs were performed using Malvern zeta sizer (Zetasizer Nano-Zs90, MPT-Z, UK). Exactly, 1 ml was taken from each formulated nanodispersion and diluted with deionized water prior to examinations to confirm that the light scattering intensity is in the instrument’s sensitivity range (Wang et al., 2012). Results were the average of three measurements.

#### Entrapment efficiency percent (EE%) determination

The prepared MTD-SLNs dispersions were centrifuged using a cooling centrifuge (2-16KL, Sigma Laborzentrifugen GmbH, Osterode am Harz, Germany) at 12,000 rpm for 2 h at 4 °C (Badawi et al., 2018). The supernatant was collected to measure the free drug concentration using ultraviolet (UV) spectrophotometer (V-630, Jasco, Tokyo, Japan) at 277 nm (Nohemann et al., 2017). The entrapment efficiency percent EE% was calculated according to the following equation (Badawi et al., 2018):

$$\mathrm{EE}\%=\;\frac{\text{W initial drug}-\text{ W free drug}}{\text{W initial drug}}\;\times 100$$

‘W initial drug’ is the amount of initial drug used for the assay

‘W free drug’ is the amount of free drug measured in the supernatant

#### Transmission electron microscope (TEM)

Morphology of the prepared MTD-SLNs was detected via transmission electron microscopy (Model JEM-1230, Jeol, Tokyo, Japan). One drop of the diluted sample was applied to a copper grid coated with carbon film after being stained with phosphotungstic acid (1%; w/v) as a negative stain (Montenegro et al., 2012).

#### *In vitro* drug release studies

The in vitro release of MTD from all developed formulae of MTD-SLNs was performed by the dialysis bag technique (Gönüllü et al., 2015) in dissolution media consisting of phosphate buffer solution pH 4.5 (Yang et al., 2019; Albash et al., 2020). A certain amount of MTD – SLNs dispersion was measured and transferred to the bags which were then immersed in the dissolution media. The temperature was controlled via a shaking water bath (WSB-18, Daihan Scientific Co. Ltd., Gangwon, South Korea) at 37 °C and shaken at 100 rpm. The precise volume of the samples was withdrawn at regular time intervals till 24 h and replaced with an equal volume of fresh dissolution media. The cumulative release of MTD from lipid nanoparticles was determined spectrophotometrically at 277 nm (Nohemann et al., 2017).

#### Selection of the optimized formula

The optimized formula was selected after data analysis by Design Expert^®^ software. This was done via applying the constraints on all independent variables to obtain particles with the least PS and PDI as well as high ZP and EE% in addition to sustained cumulative % drug release. The suggested optimized formula was prepared and characterized.

#### Formulation of MTD-SLNs vaginal emulgel

The optimized formula of MTD-SLNs was selected for the preparation of vaginal emulgel. To prepare the emulgel formulations, the emulgel forming polymer (Carbopol 934) was dispersed in deionized water in a concentration of 1% and mixed for 10 min. Subsequently, an accurate amount of the optimized MTD-SLNs dispersion was added and stirred using a high-speed stirrer at almost 1000 rpm for 30 min then the addition of Triethanolamine as a pH adjusting agent was done, to yield emulgel with good consistency containing a final concentration of 0.75% MTD (Kesharwani et al., 2016). As well, 0.75% MTD gel formulation (without nanoparticles) was prepared to be evaluated and compared with the optimized MTD-SLNs emulgel formulation.

### Characterization of MTD-SLNs vaginal emulgel

#### Physical examination, spreadability, and pH determination

The MTD-SLNs emulgel formula in addition to the MTD gel formula was inspected visually for their homogeneity color, syneresis (mean expulsion of a liquid from an emulgel). In addition, spreadability was examined via placing 0.5 g emulgel inside a pre-marked circle of 1 cm diameter on a glass plate over which another glass plate was placed. A mass of 500 g was left to rest on the upper glass for 5 min. then the increase in the diameter was measured (Wang et al., 2012). In addition, for pH determination, exactly one gram of the formulated MTD-SLNs emulgel was dispersed in 100 ml of water. The pH was then measured using a pH meter (JENWAY 350, UK) (Madan et al., 2014).

### Determination of the drug content

One gram from MTD-SLNs emulgel or MTD gel was dissolved in 50 ml of ethanol using a standard volumetric flask. Following a 1 ml of 50 ml volumetric flask was diluted in a 10 ml volumetric flask. The amount of drug for each one gram gel was detected spectrophotometrically at 277 nm (Nohemann et al., 2017) and the drug content was calculated using the calibration curve (Wang et al., 2012).

### Rheological studies

The viscosity of the prepared MTD-SLNs emulgel and the MTD gel was measured using Brookfield viscometer (DV-II Pro Viscometer, Boston, MA, USA) with spindle (T-D). The sample was exposed to continuous speed rate variation upward from 1 to 100 s^−1^ then backward from 100 to 1 s^−1^ at 25 ± 1.0 °C and the examined viscosity was recorded (Mekkawy et al., 2013).

### *In vitro* release studies

The in vitro release of MTD from the prepared emulgel formula in comparison to the MTD gel formula was performed by the dialysis bag technique. A specific amount of MTD-SLNs emulgel formulae was added to the presoaked dialysis bag. Subsequently, the bag was immersed in the dissolution media; then the experiment was accomplished as aforementioned (Cassano & Trombino, 2019). The release profile of MTD was examined by fitting the experimental data to equations that describe different kinetic orders namely zero order, first order, and Higuchi diffusion kinetics. Based on the high regression coefficient value the kinetic order was selected.

### Clinical study of the MTD-SLNs vaginal emulgel

#### Setting and ethical approval

Patients’ selection was accomplished in the Obstetrics & Gynecology Department, Faculty of Medicine, Alzahraa University Hospital. Sixty married females who complained from BV and aged between 18 and 44 years (reproductive age) were chosen for the study. Moreover, essential ethical permission was obtained from the ethical committee office code number: 202009379 Al-Azhar University, as well as written informed consent, which was essential for participation in the study. Conduction of the study was done in agreement with the ethics that have their origin in the Declaration of Helsinki Good Clinical Practice. The staff notified the participants with the aims, dates, treatments, diet, probable risks, and general activities during the clinical part of the study.

#### Patients

The current study included sixty female patients who suffered from one or more of the following: grayish-white vaginal discharge, malodor, itching, dyspareunia, and abdominal pain, then confirmed to be having BV via diagnosis using Amsel’s criteria and Gram stain. The exclusion criteria were pregnancy, lactation or taking antibiotics, immunosuppressive, anti-parasitic, anti-coagulant, or vaginal drugs during the last 14 days, and using an intrauterine device. Together with having a history of known medical diseases, abnormal uterine bleedings, having cervicitis, and cervical intraepithelial neoplasia. Lastly, any women with Trichomonas or Candida in their samples were excluded from this study cases.

#### Study design

The recruited patients were subjected to complete history taking which includes: a personal history (age, occupation, and education), menstrual history (last menstrual period), and obstetric history (parity and contraceptives).

BV was confirmed at baseline during pelvic checkup using Amsel’s criteria and Gram stain. At least 3 out of 4 Amsel’s criteria must be fulfilled to make a diagnosis of BV (Chavoustie et al., 2015). They include:

- Vaginal pH more than 4.5.
- Fishy smell release upon addition of alkali (10% potassium hydroxide).
- Characteristic thin, grayish-white discharge on examination.
- Occurrence of clue cells on microscopy of vaginal fluid mixed with normal saline.

Accordingly, any signs of vaginitis and abnormal discharges were detected and examined vaginally via a sterile speculum. The pH of the vaginal secretions was examined by placing a sample from the lateral wall of the vagina on pH paper. Samples of vaginal secretion were taken from the upper-lateral side of the vaginal wall via a sterile swab. Afterward, the samples were placed onto three different slides. Normal saline was added to the first followed examination by (X_10_) and (X_40_) objective lenses of the microscope for the presence of squamous epithelial cells with adherent bacteria ‘clue cells.’ While, a drop of 10% KOH solution was added to the second specimen to perform the Whiff test (potassium hydroxide release to the vaginal fluid) and to assess amine (fishy) odor, and inspected microscopically for the probable occurrence of Candida (hyphae or mycelia). Eventually, the third specimen was used for Gram stain and BV diagnosis. Gram stains were prepared by spreading the vaginal secretion by a sterilized bacteriological loop on a clean slide and allowing it to dry in air and fixed by passing the slide with the smear side uppermost over the flame for 2–3 times to heat-fix smears to the slides. The smear was covered with crystal violet, the primary stain, for 20 s followed by gentle rinsing off the stain with water. The smear was covered with Gram’s iodine, the mordant, for 1 min followed by pouring off the excess Gram’s iodine. The acid-alcohol decolorizer was run over the smear until the solution appeared clear followed by gentle rinsing with water. The smear was covered with diluted basic fuchsin, the counterstain, for 20 s followed by gentle rinsing with water and placing slides at an angle to air dry. The dried smear-slide was examined by oil immersion (X_100_) objective lenses of the microscope for squamous epithelial cells with adherent coccobacilli ‘clue cells’ (Chavoustie et al., 2015; Zare et al., 2019).

This study is a randomized clinical study. Eligible patients were randomly assigned into two groups each one consisting of 30 women, group (A) and group (B). Group A received the MTD-SLNs vaginal emulgel (0.75%), while group B received the marketed product commonly used in the treatment of BV (Metron^®^), vaginal gel (0.75%). The patients were required to use the assigned treatment once daily at bedtime for five consecutive days and to avoid vaginal douching/over washing. The women reported for examination on day 3 and then at the end of treatment on day 5. Also, a follow-up for the patients after 3 months to check for recurrence was performed.

In the follow-up visits, the whole diagnosis procedures were repeated and the BV treatment were considered effective if no Amsel’s criteria and negative Gram stain results were noticed. All the data were collected and statistically analyzed.

### Statistical analysis

For the clinical study part: The statistical analysis was done using SPSS statistical software (version 20.0). Chi-square was used to evaluate the significance of the difference between quantitative variables in addition *p* ≤ .05 was considered to be statistically significant while *p* ≤ .01 was considered to be highly significant. The data are displayed as the mean ± standard deviation.

## Results and discussion

### Factorial design optimization

**Figure 1.**
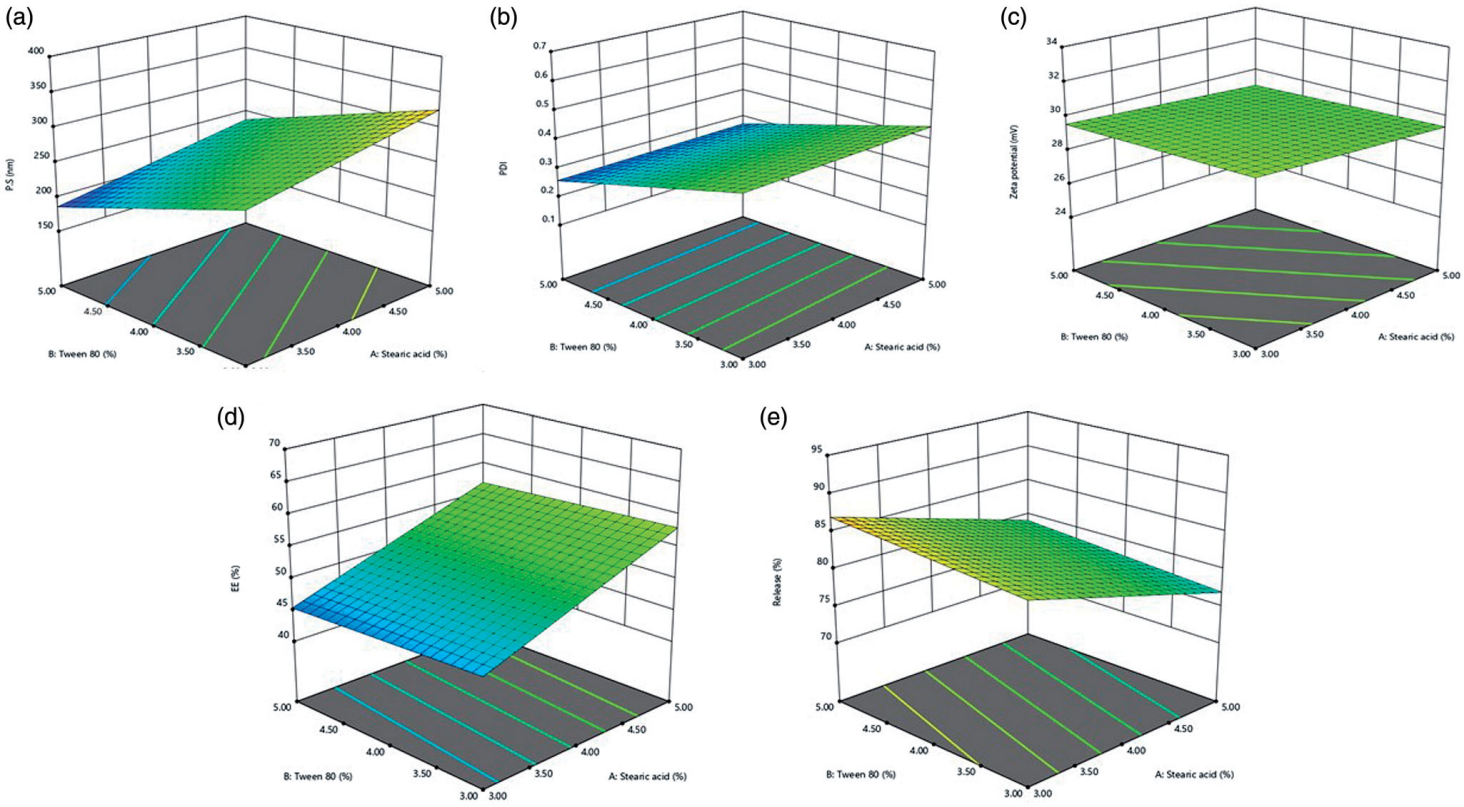
Three-dimensional response surface plots for the impact of the investigated variables on (a) particle size, (b) PDI, (c) zeta potential, (d) entrapment efficiency %, and (e) cumulative drug release %.

**Figure 2.**
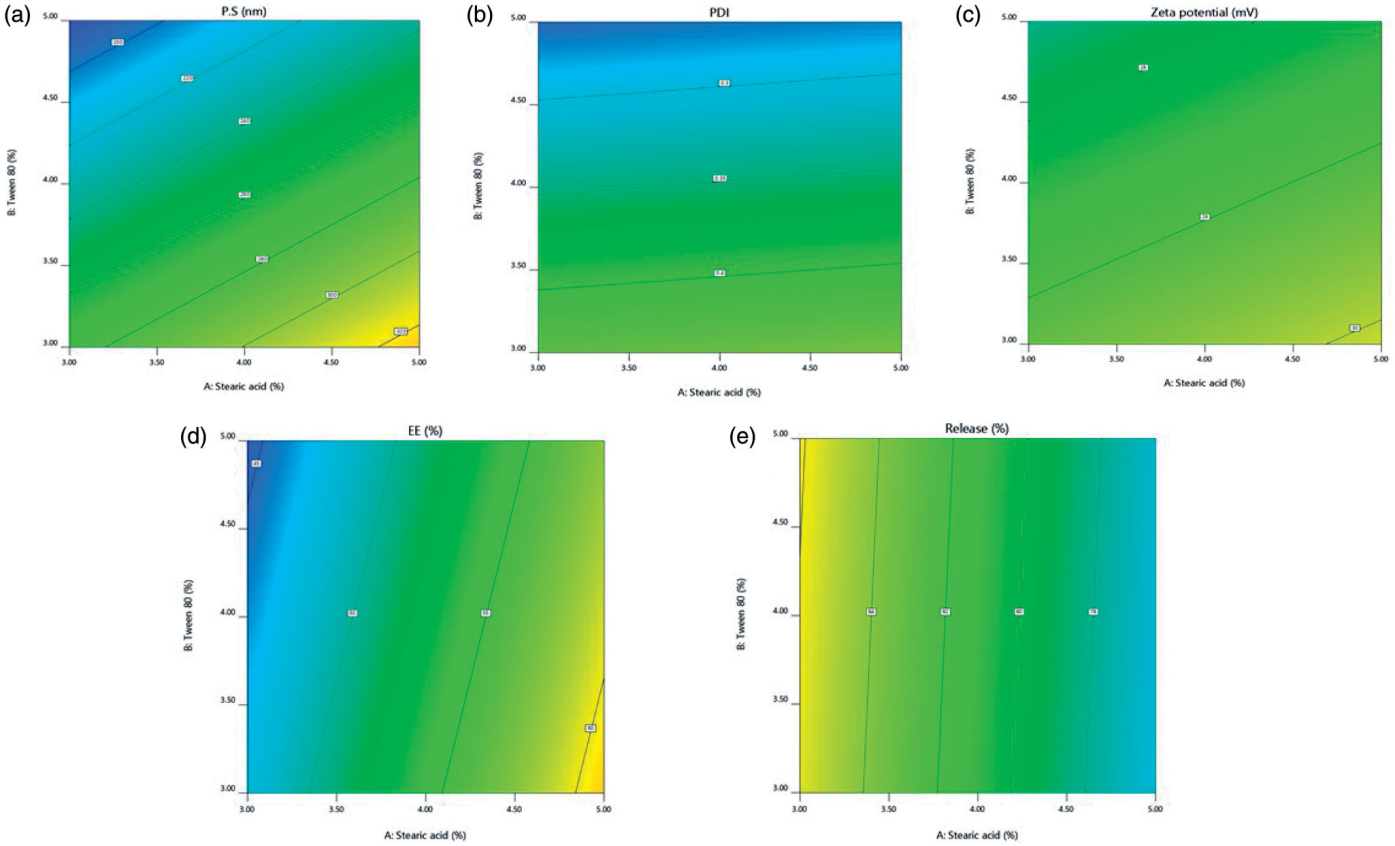
Contour plots for the impact of the investigated variables on (a) particle size, (b) PDI, (c) zeta potential, (d) entrapment efficiency %, and (e) cumulative drug release %.

The obtained results are shown in Table 2. Concerning, the design analysis results in Table 3, the selected model was two-factor interaction and it was observed that the predicted *R*^2^ values were in agreement with the adjusted *R*^2^ values in all studied responses (within approximately 0.2 of each other) except in PDI and ZP. The adequate precision value of the model usage is for verifying its adequacy to navigate the design space in which a ratio greater than 4 is considered to be desirable (Albash et al., 2021) and that was detected in all responses as presented in Table 3. To demonstrate the effect of the independent variables and their interactions on the dependent ones, three-dimensional response surface plots (Figure 1) and contour plots (Figure 2) were constructed.

**Table 2.** Composition and results of the 2^3^ full factorial design for formulations of MTD loaded SLNs.

Formulae code	Variables			Responses				
	X_1_	X_2_	X_3_	Y_1_*	Y_2_*	Y_3_*	Y_4_*	Y_5_*
	(%)	(%)	(%)	(nm)		(mV)	(%)	(%)
SLN1	5.00	5.00	50.00	230 ± 9.7	0.24 ± 0.11	−25.9 ± 3.6	58.7 ± 4.9	79.5 ± 5.1
SLN2	3.00	3.00	50.00	225 ± 7.2	0.29 ± 0.02	−29.0 ± 1.9	48.6 ± 5.7	89.1 ± 4.6
SLN3	3.00	3.00	100.00	311 ± 11.3	0.57 ± 0.13	−30.9 ± 4.4	42.5 ± 3.1	81.2 ± 3.9
SLN4	3.00	5.00	100.00	209 ± 5.4	0.28 ± 0.08	−28.5 ± 5.1	44.3 ± 5.9	82.7 ± 6.5
SLN5	5.00	5.00	100.00	231 ± 6.9	0.30 ± 0.07	−32.1 ± 2.4	52.8 ± 2.1	72.5 ± 2.8
SLN6	5.00	3.00	50.00	301 ± 13.3	0.48 ± 0.19	−28.8 ± 5.6	65.6 ± 3.4	81.4 ± 6.1
SLN7	5.00	3.00	100.00	362 ± 8.5	0.40 ± 0.09	−30.1 ± 2.3	60.5 ± 7.1	71.7 ± 3.1
SLN8	3.00	5.00	50.00	175 ± 10.6	0.22 ± 0.05	−25.0 ± 4.5	48.1 ± 6.8	90.5 ± 5.3

*Results are expressed as the mean of 3 replicates ± SD.

SLNs: solid lipid nanoparticles; X_1_: lipid concentration; X_2_: surfactant concentration; X_3_: sonication amplitude; Y_1_: particle size; Y_2_: polydispersity index; Y_3_: zeta potential; Y_4_: entrapment efficiency; Y_5_: cumulative % drug release.

**Table 3.** Results of regression analysis of the responses.

Response	Adequate precision	*R* ^2^	Adjusted *R*^2^	Predicted *R*^2^	*p*-Value
Y_1,_ Particle size	10.98	0.91	0.85	0.66	.0126
Y_2_: Polydispersity index	4.14	0.68	0.45	−0.25	.163
Y_3_: Zeta potential	5.05	0.73	0.52	−0.09	.127
Y_4_: Entrapment efficiency	10.46	0.93	0.86	0.71	.012
Y_5_: Cumulative % drug release	25.37	0.98	0.97	0.94	.0003

### Impact of formulation variables on different studied responses

PS of the fabricated MTD-SLNs was within the nano-range from 175.5 to 362.9 nm, as displayed in Table 2. ANOVA analysis demonstrates that both X_1_ and X_2_ had a significant impact on the PS of the fabricated MTD-SLNs with *p*-values equal to .0385 and .0063, respectively. It was found that there was a positive relation between lipid concertation (X_1_) and PS where increasing the concentration of lipid resulted in particles with large size. This may be due to the reduced sonication efficiency at higher lipid contents because of high sample viscosity, which led to larger particles (Hosny, 2016). Moreover, deficiency of adequate surfactant to surround the particle surface is the probable cause for large particle size (Shah, Eldridge, et al., 2014). Furthermore, high lipid contents led to high particle concentration that increases the possibility of particle contact and aggregation (Freitas & Müller, 1998). While surfactant concentration (X_2_) had an inverse relation with PS where increasing the concentration of surfactant resulted in smaller PS of the prepared MTD-SLNs. This could be described by the reduced interfacial tension between lipid and aqueous phases when the concentration of surfactant increased, thus regulate the aggregation of the particles and resulting in lower particle size (Mehnert & Mäder, 2001). Moreover, as previously stated, particle surface stabilization can occur with higher surfactant concentration which develops a steric barrier on the lipid matrix surface, hence preventing aggregation (Harivardhan Reddy & Murthy, 2005).

Regarding PDI, a low value approaching zero illustrates a perfectly homogenous dispersion with a narrow PS range. While, a high value approaching one represents a highly polydisperse dispersion (Albash et al., 2021). All variables showed no significant effect with *p*-values of .83, .055, and .28 for (X_1_), (X_2_), and (X_3_), respectively. The PDI of the measured MTD-SLNs ranged from 0.22 to 0.57 which showed that the prepared formulations showed good homogeneity (Table 2) (Badawi et al., 2018).

The stability of nanoparticles is commonly indicated by ZP which is employed to measure the nanoparticles’ charge (Badawi et al., 2020). ZP of the developed formulations ranged from −25.0 to −32.1 mV (Table 2). The prepared formulations exhibited negative zeta potential due to the occurrence of terminal carboxylic groups in the lipids (Badawi et al., 2020). It is worth stating that there was a small difference in ZP values, but the variation was not significant.

EE% refers to the amount of drug, expressed as a percentage, entrapped by the nanoparticles in comparison to the amount of drug added (Shah, Malherbe, et al., 2014). The capability of the formulated nanoparticles to accommodate a sufficient amount of drug is a vital constraint for its possible application as a drug delivery system. The entrapment efficiency of the prepared MTD-SLNs formulations varied from 42.5% to 65.6% (Table 2). ANOVA results revealed that X_1_ only had a significant impact on the EE% of the fabricated MTD-SLNs with *p*-value equal to .0031. A positive effect with increasing the lipid concentration (X_1_) was detected. This could be due to that higher lipid amount offers additional space in order to encapsulate an extra amount of drug during SLNs preparation. In addition, it leads to a decrease in the rate of drug diffusion into the external phase due to higher lipid phase viscosity and accordingly exhibited higher entrapment efficiency (Hosny, 2016).

**Figure 3.**
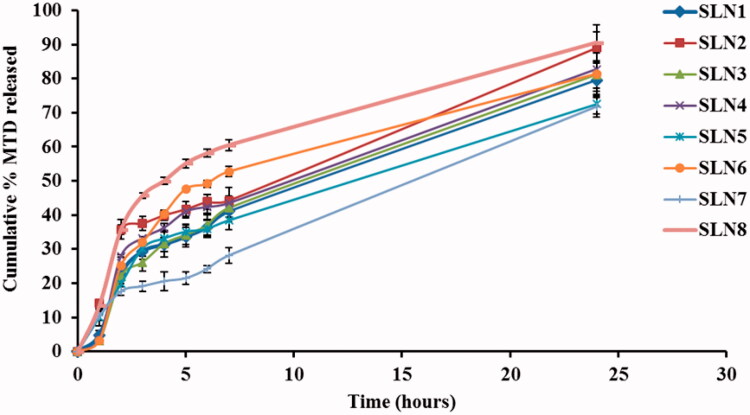
*In vitro* release of MTD from the prepared SLNs through cellulose membrane over 24 h. MTD, metronidazole; SLNs, solid lipid nanoparticles.

The in vitro release profile of MTD-SLNs formulations over 24 h is displayed in Figure 3 in which a biphasic release pattern was obvious. This behavior has been stated for SLNs (Nabi-Meibodi et al., 2013). Burst drug release is probably due to the drug attached to the nanoparticles’ surface, whereas sustained release is regularly due to the entrapped drug in the lipid core (Tran et al., 2014). Cumulative MTD release over 24 h ranged from 71.7% to 90.5% as shown in Table 2. ANOVA results show that both X_1_ and X_3_ had a significant impact on the cumulative MTD release of the fabricated MTD-SLNs with *p*-values equal to .0002 and .0003, respectively. X_1_ had a negative influence on the MTD release which could be clarified by the reduced surface area due to large particle size thus, decreasing the release rate (Mainardes & Evangelista, 2005). Moreover, increasing the lipid concentration leads to higher viscosity and more rigid solidified nanoparticles which may also delay the diffusion of the drug to the dissolution medium (Emami et al., 2015). Also, X_3_ had a negative impact on the MTD release as it led to the formation of large particles and hence small surface area obtainable for dissolution medium so led to retarded release (Venkateswarlu & Manjunath, 2004).

### Morphology examination

**Figure 4.**
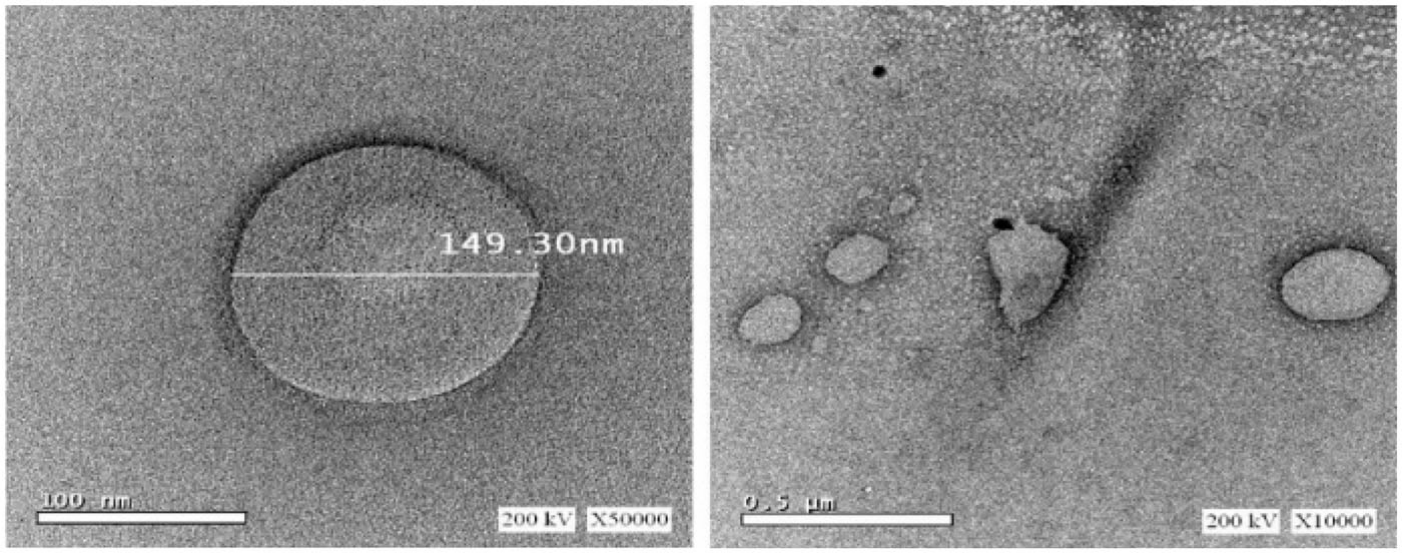
Morphology of MTD-SLNs obtained from batch SLN8. MTD, metronidazole; SLNs, solid lipid nanoparticles.

Figure 4 revealed that the transmission electron micrograph of the MTD-SLNs samples presented spherical particles with no aggregation. We reported only one image as an example because all images attained were alike.

### Selection of the optimized MTD-SLNs formulation

To select the optimized formula, certain conditions were set in Design Expert software version 12. These conditions favored nanoparticles with the highest EE%, cumulative % drug release, ZP, and lowest PS as well as PDI. The optimized formula which met these criteria was found to be composed of stearic acid as a lipid 4%, tween 80 as a surfactant 4%, and using a sonication amplitude of 75%. The observed responses of MTD-SLNs optimized formula were EE% of 52%, cumulative % drug release after 24 h of 87%, a PS of 256 nm, a PDI of 0.35, and a ZP of −29.5 mV, which are in close agreement with the predicted values.

### MTD-SLNs vaginal emulgel

To maximize the benefit of the MTD-SLNs optimized formula, it was incorporated into emulgel structure using Carbopol 934 emulgel forming agent in order to enhance the application and vaginal retention of the drug.

### Physical examination, spreadability, pH determination, and drug content

The prepared optimized MTD-SLNs emulgel, as well as the MTD gel formulae, were white with a smooth and homogenous appearance. One of the important formulation parameters for vaginal compatibility is the pH (Rençber et al., 2017). The pH values were in the acceptable range, as per the pH of the vagina (around 3.5–4.5) with the purpose of avoiding any vaginal irritation upon application, which indicates the suitability of the formulations for the vaginal application (Mudhney et al., 2015). Results of the pH are shown in Table 4.

**Table 4.** Physical characters of prepared vaginal formulations.

Formula Code	Color	Homogeneity	Texture	pH*	Spreadability* (cm)	Drug Content* (%)
G1	White	Homogeneous	Smooth	4.94 ± 0.12	7.41 ± 0.10	99.30 ± 0.58
G2	White	Homogeneous	Smooth	5.11 ± 0.06	6.52 ± 0.21	98.99 ± 0.63

*Results are expressed as the mean of 3 replicates ± SD.

G1: MTD-SLNs emulgel formula; G2: MTD gel formula.

Spreadability of the formulation is a vital characteristic when considering patient compliance as it is responsible for precise dosage delivery to the target site and ease of application on the mucosa (Rençber et al., 2017). Formulations with high values of spreadability permit easy application and hence higher surface area obtainable for drug permeation. The prepared formulations gave an acceptable range as shown in Table 4, which indicates good spreadability by a small amount of shear (Bachhav & Patravale, 2010). In addition, the prepared formulae exhibited an acceptable range of drug content and low standard deviations (Table 4). It confirms that the drug is reliably dispersed in the vaginal formulations.

### Rheological properties

The rheological property of the vaginal semisolid formulations affects their potential for vaginal application in a fundamental way because the shear rate on the preparation is high during vaginal application. If the viscosity is too high, this will lead to difficulty in application and thus irritation. On the other hand, if it is too low, it will result in increased drainage (Mudhney et al., 2015). Therefore, the formulation should have an optimum viscosity for easy application to the vaginal mucosa. Commonly, viscosity values in an optimum range enhance the retention time & mucoadhesive property. The viscosity values were 90,500 and 50,346 cp at speed 5 rpm for G1 and G2, respectively. All prepared formulae exhibited non-Newtonian pseudoplastic flow with shear-thinning behavior as well as thixotropy, representing that the disarranged viscosity of the system decreases with the increase in shear rate (Yao & Patel, 2001). On the basis of rheological studies, the prepared formulations showed good viscosity and spreading on vaginal application and the results are in agreement with Mudhney et al. (Mudhney et al., 2015).

### In vitro release studies

**Figure 5.**
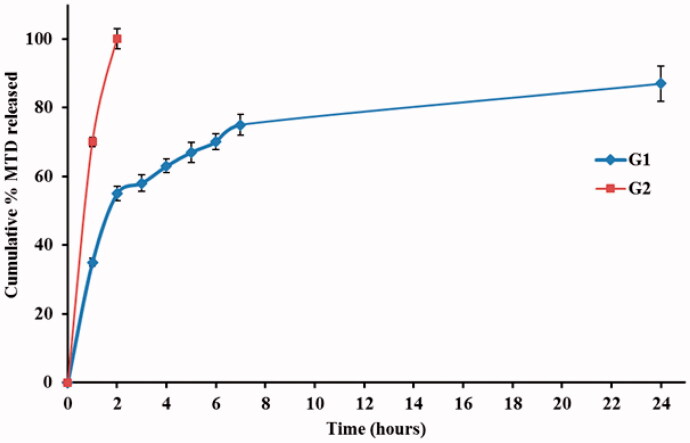
*In vitro* release of optimized MTD-SLNs vaginal emulgel (G1) in comparison to MTD gel (G2) formulations through cellulose membrane over 24 h. Values are presented as means ± SD.

The release study of MTD from vaginal formulations was performed using the dialysis bag membrane technique (Cassano & Trombino, 2019). In vitro release profiles of MTD from G1 and G2 in phosphate buffer solution pH 4.5 were displayed in Figure 5. It was observed from the obtained data that, the release rates of G1 and G2 formulations after 2 h were 55% and 100%, respectively. While the release rate of MTD from G1 at the end of 24 h reached 82%. This was reinforced by the fact that the conventional gel formulation of MTD (G2) exhibited fast release of the drug which is in accordance with Maru et al (Maru et al., 2012). On the other hand, MTD-SLNs emulgel formulation (G1) showed a prolonged release rate which may be explained by the difference in viscosity of the formulations where the release amount of the drug decreases as the viscosity increases (Rençber et al., 2017). Furthermore, the investigated formulation (G1) demonstrated release characteristics following Higuchi kinetics. This result recommended that the primary release mechanism of MTD from the lipid matrix and the emulgel is diffusion.

### Clinical study of the MTD-SLNs vaginal emulgel

The clinical study was done to evaluate the prepared MTD-SLNs vaginal emulgel in comparison with the market product Metron^®^ (vaginal gel) which is commonly used in the treatment of BV. Sixty female patients were enrolled successfully for 5 days in this study to evaluate the treatment of BV and the patient’s demographics were recorded as shown in Table 5. The diagnosis was clinically performed based on Amsel’s criteria and verified via microbiological examination. Every 30 patients were randomly enrolled in a group; Group A received MTD-SLNs vaginal emulgel and Group B received the market product Metron^®^ vaginal gel. The treatments were applied once daily at bedtime for 5 days. Firstly, baseline signs, symptoms, and evaluation were taken for each patient to register the pretreatment information as shown in Table 6 to be able to assess the clinical efficacy and safety of the treatment regimen. In addition, the patients in each group were followed up by clinical and microbiological examination at day 3 as shown in Table 7, and at the end of the treatment (day 5) as shown in Table 8. Furthermore, the patients were followed up for any sign of allergy and after 3 months of treatment to investigate the incidence of recurrence infection (Table 9). The contributors showed good acceptance of the formulations and stated no adverse feedbacks while using them or afterwards.

**Table 5.** Demographic data of the studied groups.

Variables	Group A	Group B	Chi-square	
	*n* = 30	*n* = 30		
	Mean ± SD	Mean ± SD	*t*	*p*-Value
Age (years)	40.21 ± 1.12	41.23 ± 2.03	1	.737
BMI (kg/m^2^)	29.12 ± 1.63	28.71 ± 1.45	1	.648
Occupation				
House wife	20 (66.66%)	19 (63.33%)	1	.732
Worker	10 (33.33%)	11 (36.66%)	1	.521
Education				
Primary	10 (33.33%)	9 (30%)	1	.652
Secondary	12 (40%)	15 (50%)	3	.246
Collage	8 (26.67)	6 (20%)	2	.547
Parity	4.23 ± 2.1	3.4 ± 5.2	1	.683

**Table 6.** Comparison between the studied groups before the start of the treatment.

Amsel’s criteria	Group A		Group B		Chi-square	
	*n*	%	*n*	%	*t*	*p*-Value
PH > 4.5	30	100%	30	100%	1	.837
Vaginal discharge	28	93.33%	27	90%	1	.672
KOH + ve (Wife test)	26	86.67%	28	93.33%	1	.715
Malodor	30	100%	30	100%	2	.837
Itching	26	86.67%	28	93.33%	2	.574
Dyspareunia	25	83.33%	24	80%	1	.626
Abdominal pain	20	66.67%	21	70%	1	.635
Gram stain + ve	30	100%	30	100%	1	.837

**Table 7.** Comparison between the studied groups at day 3 of treatment.

Amsel’s criteria	Group A		Group B		Chi-square	
	*n*	%	*n*	%	*t*	*p*-Value
PH > 4.5	8	26.67%	15	50%	19	.003*
Vaginal discharge	5	16.67%	11	36.67%	17	.003*
KOH + ve (Wife test)	2	6.67%	5	16.67%	2	.143
Malodor	5	16.67%	10	33.33%	16	.042*
Itching	4	13.33	12	40%	22	.000*
Dyspareunia	9	30%	10	33.33%	17	.542
Abdominal pain	8	26.67%	10	33.33%	19	.412
Gram stain + ve	4	13.33%	10	33.33%	17	.002*

*Significant difference between the results of both groups.

**Table 8.** Comparison between the studied groups at day 5 of treatment.

Variables	Group A		Group B		Chi-square	
	*n*	%	*n*	%	*t*	*p*-Value
PH > 4.5	3	10%	5	16.67%	1	.243
Vaginal discharge	2	6.67%	5	16.67%	2	.143
KOH + ve (Wife test)	2	6.67%	2	6.67%	1	.841
Madodor	2	6.67%	2	6.67%	1	.832
Itching	1	3.33%	1	3.33%	1	.826
Dyspareunia	2	6.67%	4	13.33%	1	.625
Abdominal pain	3	10%	3	10%	1	.782
Gram stain + ve	2	6.67%	4	13.33%	1	.427

**Table 9.** Incidence of complication of the treatment in both groups.

Variables	Group A		Group B		Chi-square	
	*n*	%	*n*	%	*t*	*p*-Value
Allergy	2	6.66%	4	13.33%	2	.583
Recurrence of infection of the treatment in 3 months	2	6.66%	10	33.33%	17	.001*

*Significant difference between the results of both groups.

The study results showed no significant (*p* > .05) difference between the two examined groups in terms of demographic, as age, occupation, level of educations, and parity (Table 5). There is no significant difference between both groups before treatment as regards Amsel’s criteria, symptoms and gram stain results (Table 6). After 3 days of treatment, there was a clinical improvement of symptoms of the patients from both group A and group B. However, the improvement in group A was much better as there was a significant decrease in Amsel’s criteria; where the pH did not change in only 26.67% in group A while 50% in group B and the vaginal discharge was present in only 16.67% of the patients receiving MTD-SLNs emulgel in comparison to 36.67% in the group receiving Metron. There was also a significant enhancement in the symptoms of women receiving MTD-SLNs emulgel in contrast to the group receiving market product where the cases still complained of malodor 16.67% to 13.33% and itching still present in 13.33% to 40% respectively in group A to group B respectively (Table 7). On the other hand, although there was a reduction in the dyspareunia and abdominal pain, it was not significant. As for the gram stain under an electron microscope at the start of the study 100% of both groups were positive (*n* = 30 per group), upon receiving the treatment for only 3 days most of the group receiving MTD-SLNs emulgel showed negative results; only 4 patients remained positive (13.33%), while 10 patients remained positive (33.33%) in group B, which was statistically highly significant.

**Figure 6.**
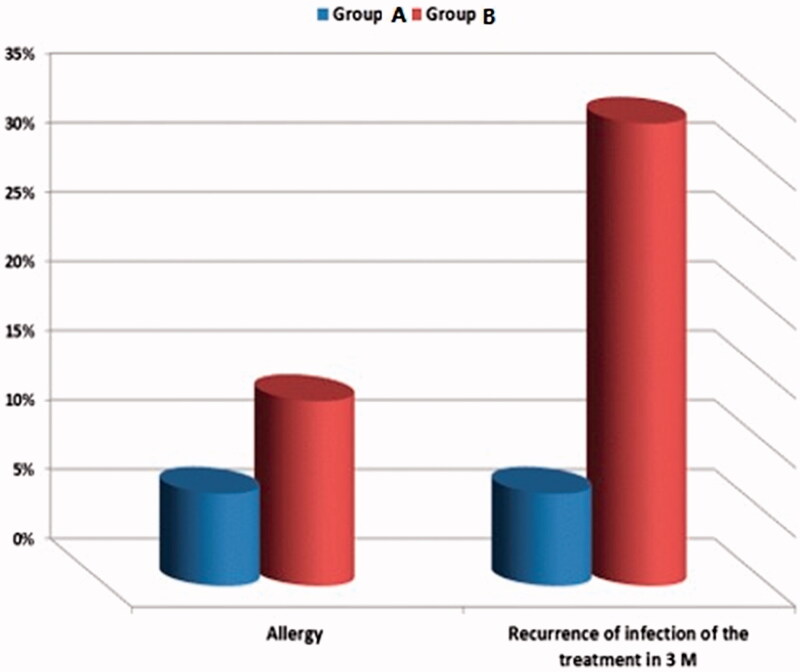
Incidence of complication of the treatment in both groups.

At the end of the treatment day 5, the patients in both groups showed improvement, yet there was no significant difference between the groups in regard to Amsel’s criteria, symptoms, and gram stain (Table 8). The incidence of complications was higher in group B than in group A as the cases with allergy equals 13.33% in group B and 6.6% in group A, however, the variation between the studied groups is not statistically significant (*p* > .05). Nevertheless, the incidence of recurrence of infection within 3 months of treatment was significantly lower (*p* = .001) in group A (16.66%) compared to 33.33% of group B (Table 9 and Figure 6). Pertaining to the previous, the total number of patients that were cured after only 3 days of treatment using MTD-SLNs emulgel was significantly higher than those using the market product, which showed that a shorter period of treatment was required.

In our study, vaginal MTD-SLNs emulgel was observed to be faster and more effective in treatment than vaginal Metron gel in the BV cases. The majority of the cases that were treated by vaginal emulgel seemed to be pleased with its quick cure (after 3 days of application) and a major decrease in the recurrence rate compared to the generally used commercial vaginal gel. Moreover, there was no irritation or significant adverse effects with the vaginal MTD-SLNs emulgel.

Generally, treatment against BV emerged with MTD as the drug of choice for its action against several anaerobic organisms. But, regrettably, the cure rates associated with MTD ranged from 50–80%, with a high recurrence rate (Schwebke & Desmond, 2011). One of the main reasons for treatment failure is vaginal biofilm which plays a key role not only in BV pathogenesis but also in its recurrence (Tomás et al., 2020). Bacterial biofilms are established when an accumulation of adherent microorganisms occurs on a living surface (Malaekeh-Nikouei et al., 2020). The biofilm’s structure avoids the antimicrobial penetration in the matrix as well as the direct contact with the microorganisms. Concurrently, an increase in antibiotics resistance is detected, which leads to treatment failures (Tomás et al., 2020). Several studies recommend the lack of effectiveness of conventional antimicrobial treatments and high recurrence rates of BV because of their failure to destroy the vaginal biofilm in addition to their adverse effects on healthy vaginal microflora (Tomás et al., 2020).

The antimicrobial agents’ encapsulation into lipid-based carriers has many advantages such as deactivation resistance, as well as reduction of adverse effects and systematic toxicity. Furthermore, the prolonged-release behavior of antibiotics from the lipid nanoformulations was reported in biofilm eradication and improved antibacterial activities (Malaekeh-Nikouei et al., 2020). According to several studies, SLNs were found to have potential antibiofilm activity against many types of bacteria (Singh et al., 2014; Islan et al., 2016; Sharma et al., 2016; Akhtari et al., 2020). SLNs serve as a coating agent for the antibacterial drug thus do not allow the bacterial adhesion to the surface of the formulation and therefore preventing bacterial biofilm formation (Taylor et al., 2014). Simultaneously, the nano size of SLNs enables the drug penetration inside the cells and hence destroys the organism successfully (Singh et al., 2014).

Accordingly, these results highlight the effectiveness of the therapeutic value of the optimized MTD-SLNs emulgel formulation over the commercial formulation.

## Conclusion

BV is one of the major vaginal infections in women of reproductive age but the traditional treatment has a high most recurrence rate. So, in this study, we successfully developed vaginal emulgel of MTD loaded in SLNs optimized formula. Clinically, MTD-SLNs vaginal emulgel had a significant therapeutic effect against BV symptoms according to Amsel’s criteria in comparison to the commercial product as well as low recurrent rate. Therefore, the abovementioned results proved that the optimized formulation could be a potential delivery system for vaginal application of MTD.
